# Prospective comparative analysis from pre‐ to postoperative changes in Single Assessment Numeric Evaluation (SANE) for patellofemoral instability

**DOI:** 10.1002/jeo2.70762

**Published:** 2026-05-29

**Authors:** Felix Zimmermann, Julian Flügel, Linda Biester, Sebastian Gebhardt, Paul Alfred Grützner, Emmanouil Liodakis, Peter Balcarek

**Affiliations:** ^1^ Arcus Kliniken Pforzheim Pforzheim Germany; ^2^ Department for Orthopaedics and Trauma Surgery, BG Klinik Ludwigshafen Heidelberg University Ludwigshafen Germany; ^3^ Klinik Für Hirurgie Kreiskrankenhaus Demmin Demmin Germany; ^4^ Department of Trauma, Hand and Reconstructive Surgery, Departments and Institutes of Surgery Saarland University Homburg Germany

**Keywords:** BPII 2.0, lateral patellar instability, PASS, SANE, SCB

## Abstract

**Purpose:**

Single Assessment Numeric Evaluation (SANE) has been introduced for the pre‐operative assessment of lateral patellar instability (LPI), but post‐operative evaluation is lacking. This study aimed to investigate the ability of SANE questions (SQs) to assess patients with LPI before and after surgery.

**Methods:**

120 consecutive patients with LPIs were prospectively assessed via the Banff Patella Instability Instrument 2.0 (BPII 2.0). Patients were randomised into three groups (40 each) and asked to answer 1 of 3 SQs: “How do you rate your knee joint if a completely stable kneecap means 100%?” (SQ1), “How do you rate your knee joint if complete satisfaction means 100%?” (SQ2), “How do you rate your knee joint if complete normal function means 100%?” (SQ3). At ≥24 months post‐operatively, 98 patients (81.6%) underwent reassessment with previous SQs and BPII 2.0.

**Results:**

Post‐operatively, the average BPII 2.0 was 77.7 ± 17.3 points. Mean SQ1, SQ2 and SQ3 results euqalled BPII 2.0 values and were 82.9%, 77.1% and 80.1%, respectively, without differences between groups (all *p* > 0.05). Compared with pre‐operative data, post‐operative correlations between BPII 2.0 and SQ1, SQ2, and SQ3 changed from high to low for SQ1 (*r* = 0.23; 95% confidence interval [CI], −0.13 to 0.53; *p* = 0.2), from low to moderate for SQ2 (*r* = 0.61; 95% CI, 0.32–0.79; *p* = 0.0003), and from low to high for SQ3 (*r* = 0.84; 95% CI, 0.70–0.92; *p* < 0.0001). 87 participants (88%) reached the patient acceptable symptom state (PASS), and 84 participants (86%) reported substantial clinical benefit (SCB).

**Conclusion:**

All three SANE questions reflected significant post‐operative improvement consistent with BPII 2.0 outcomes. However, correlations varied from pre‐ to post‐operative. While SQ1 previously demonstrated concurrent validity with BPII 2.0 pre‐operatively, SQ3 exhibited the strongest correlation with BPII 2.0 post‐operatively, warranting cautious interpretation given study limitations.

**Level of Evidence:**

Level II.

**Abbreviations**
BMI: body mass index
BPII 2.0: Banff Patella Instability Instrument 2.0
CI: confidence interval
DFO: distal femoral osteotomy
dTrochleoplasty: deepening trochleoplasty
LPI: lateral patellar instability
MCID: minimal clinically important difference
MOI: magnitude of improvement
MPFL‐R: medial patellofemoral ligament reconstruction
PASS: patients' acceptable symptom state
PROM: patient‐reported outcome measures
QOL: quality of life
SANE: Single Assessment Numeric Evaluation
SCB: substantial clinical benefit
SD: standard deviations
SQ: SANE questions
TTO: tibial tubercle osteotomy

## INTRODUCTION

Lateral patellar instability (LPI), a common condition in adolescents and young adults with high recurrence rates and persistent functional limitations, has prompted growing scientific interest and an increasing reliance on patient‐reported outcome measures (PROMs) to assess treatment effects accurately from the patient's perspective [4, 20, 23, 27]. Among the established PROMs, the Banff Patella Instability Instrument 2.0 (BPII 2.0) is the most thoroughly validated instrument specifically developed to assess disease‐specific quality of life in individuals with LPIs [8]. The BPII 2.0 consists of 23 items across five quality‐of‐life domains, each scored from 0 to 100 and averaged into a total score, where higher values reflect better disease‐specific quality of life [1, 28].

Building on the growing demand for efficient and pragmatic outcome measures, recent investigations have examined whether Single Assessment Numeric Evaluation (SANE) can serve as a meaningful alternative to more comprehensive PROMs [5, 17, 26]. SANE offers practical advantages, including reduced patient burden and ease of administration; however, they inherently lack the multidimensional depth of comprehensive PROMs and may be more susceptible to measurement error, limited content validity and contextual interpretation by patients. Moreover, their ability to capture complex constructs such as disease‐specific quality of life remains uncertain, particularly across different stages of treatment [5, 14].

Gebhardt et al. directly compared three differently phrased SANE questions (SQs) with the BPII 2.0 in a cohort of 120 patients with recurrent LPIs [6]. The authors showed that the question *“How do you rate your knee joint if a completely stable kneecap means 100%?”* demonstrated concurrent validity with the BPII 2.0, indicating that this specific SQ provides a clinically meaningful short‐form measure capable of reflecting disease‐specific quality of life in individuals with LPIs in a pre‐operative setting [6]. However, a post‐operative comparison of SQs and the BPII 2.0 has not yet been performed. Therefore, the purpose of this study was to investigate post‐operative SQ assessment to demonstrate comparable post‐operative patient‐reported outcomes to BPII 2.0 in surgically treated patients with patellofemoral instability. It was hypothesised that the reported pre‐operative correlations would also persist post‐operatively.

## METHODS

This prospective study obtained approval from the local ethics committee of Baden‐Württemberg, Germany (F‐2019‐070) and represents a follow‐up analysis of previously published data [6]. The pre‐operative data referenced in the current manuscript originate from the initial publication [6] and are incorporated here to contextualise the 2‐year outcomes.

Between October 2022 and March 2023, 120 consecutive patients (male/female 50/70; mean age 23.9 ± 8.0 years; mean BMI 25.3 ± 5.1 kg/m²) with patellofemoral instability presenting to a specialist clinic for joint‐preserving knee surgery were enroled. Inclusion required recurrent LPIs with at least two prior dislocations (last dislocation before > 4 weeks). Patients were excluded if they had any previous patellar stabilising surgery, osteoarthritis (Kellgren–Lawrence grade > 2), rheumatoid arthritis, infection or concomitant injuries such as ligament or meniscal tears.

All evaluations were conducted pre‐operatively and after a minimum follow‐up period of 24 months post‐operatively. For the evaluations, the included patients, who were all native German speakers, completed the validated German version of the Banff Patella Instability Instrument 2.0 (BPII 2.0) [1]. Additionally, patients were randomised into three groups of 40 patients with equal demographic data (see Table 1 in Gebhardt et al. [6]) to complete one of three German SQs alongside the BPII 2.0 evaluation. This approach has previously been shown to provide content validity [3]. For all the questions, patients could choose a numeric value between 0% and 100% to evaluate the state of their knee, where 0% indicated severe problems and 100% indicated no problems with the knee. SQ1 assesses “instability” (*“How do you rate your knee joint if a completely stable kneecap means 100%?”*), SQ2 assesses “satisfaction” (*“How do you rate your knee joint if complete satisfaction means 100%?”*), and SQ3 assesses “function” (*“How do you rate your knee joint if complete normal function means 100%?”*). Each patient completed one SQ in addition to the BPII 2.0. In addition, participants completed a dichotomous (*yes/no*) question (*“Do you consider the current condition of your knee joint to be satisfactory?”*) for assessment of patients' acceptable symptom state (PASS) and a trichotomous (*not at all/somewhat/very much*) question (*“How much has your knee joint benefited from the surgery?”*) for assessment of the substantial clinical benefit (SCB) post‐operatively.

**Table 1:** Patient demographic data and distribution of operative procedures in SQ1, SQ2 and SQ3.

	SQ1	SQ2	SQ3	*p*‐value
Age (years)	23.5 ± 7.2	22.6 ± 6.7	26.1 ± 10.2	n.s.
Sex (male/female)	16/17	13/18	12/22	n.s.
BMI (kg/m^2^)	24.2 ± 4.4	24.3 ± 4.3	24.9 ± 5.2	n.s.
Follow‐up (months)	32.0 ± 3.2	32.4 ± 3.0	33.0 ± 3.9	n.s.
MPFL‐R	18 (55%)	8 (26%)	9 (26%)	n.s.
MPFL‐R + TTO	7 (21%)	11 (35%)	14 (41%)	
MPFL‐R + dTrochleoplasty	5 (15%)	7 (23%)	4 (12%)	
MPFL‐R + DFO + TTO	1 (3%)	4 (13%)	3 (9%)	
MPFL‐R + TTO + dTrochleoplasty	2 (6%)	1 (3%)	4 (12%)	

*Note*: The mean values ± standard deviations and absolute and relative frequencies are shown.

Abbreviations: BMI, body mass index; DFO, distal femoral osteotomy; dTrochleoplasty, deepening trochleoplasty; MPFL‐R, medial patellofemoral ligament reconstruction; SQ, Single Assessment Numeric Evaluation question; TTO, tibial tubercle osteotomy.

At follow‐up, 98 patients (81.6%) were available and underwent reassessment using the same SQs completed pre‐operatively, along with the BPII 2.0. The remaining patients (*n* = 22; 18.4%) were not re‐enroled post‐operatively due to missing or updated contact information or because consent could not be obtained. The baseline characteristics of these patients did not differ significantly from those who completed the study.

### Statistics

The normality of the continuous data was assessed, and the data are presented as the means ± standard deviations (ranges). One‐way analysis of variance with a Bonferroni multiple comparison test and Chi‐squared test were used to test for significance between the groups. The correlation between the BPII 2.0 and the SQs was analysed with the Pearson correlation coefficient (*r*) with a 95% confidence interval (CI). The correlation strength was defined as very high if the *r* value was above 0.90, high if the r value was between 0.70 and 0.89, moderate if the *r* value was between 0.50 and 0.69, low if the r value was between 0.30 and 0.49, and negligible if the r value was below 0.30 [12]. Bland–Altman analysis (plots) was used to investigate biases (differences between the means) of each SQ in comparison with BPII 2.0. The minimal clinically important difference (MCID) in the BPII 2.0 score was assessed using the distribution‐based method by calculating half of the standard deviation of the baseline BPII 2.0 score [11]. All analyses were performed using GraphPad Prism (version 4; GraphPad Software, San Diego, CA, USA). The level of significance was set at a *p*‐value of 0.05.

## RESULTS

The patients' demographics and operative treatment procedures are shown in Table 1. At a mean follow‐up period of 32.5 ± 3.4 months, patients showed marked improvement, with the mean BPII 2.0 score increasing from 40.5 ± 16.8 points pre‐operatively to 77.7 ± 17.3 points post‐operatively (*p* < 0.0001). Eighty‐eight patients (90%) achieved an MCID of 8.7 points for the total BPII 2.0 score. Of the total cohort, 87 patients (88%) achieved the Patient Acceptable Symptom State (PASS) according to the dichotomous question evaluation. Specifically, 88%, 94% and 85% of patients achieved PASS in SQ1, SQ2 and SQ3, respectively. Eighty‐four patients (86%) of the total cohort reported a Substantial Clinical Benefit (SCB). The proportion of patients reporting SCB was 94%, 77% and 85% for SQ1, SQ2 and SQ3, respectively. The mean scores for SQ1, SQ2 and SQ3 increased from 44.2%, 42.6% and 44.2% to 82.9% (*p* < 0.0001), 77.1% (*p* < 0.0001) and 80.1% (*p* < 0.0001), respectively, with no significant differences observed between the groups (all *p* > 0.05).

**Figure 1:**
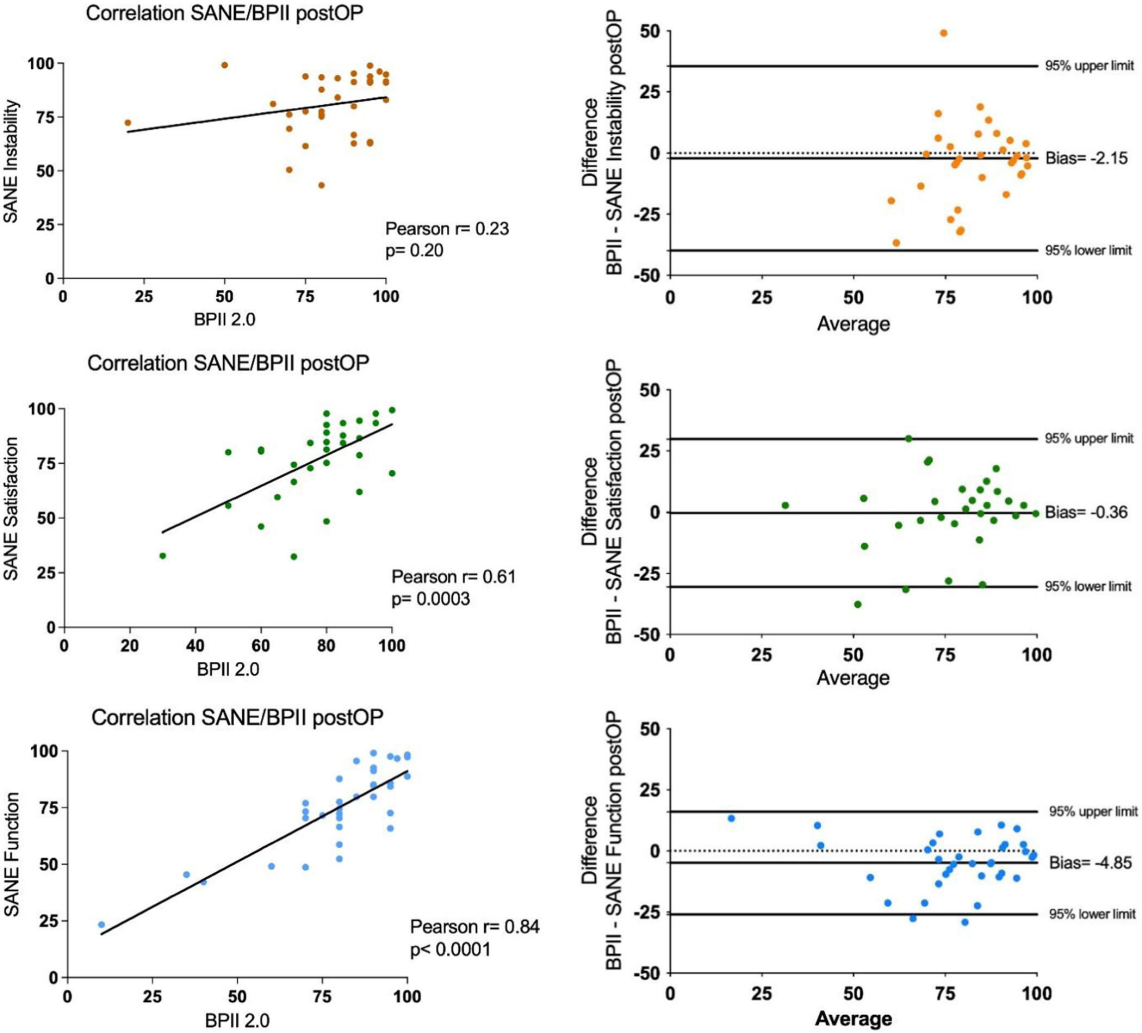
Pearson correlation and Bland–Altman plots of (a) SQ 1 (Instability), (b) SQ 2 (Satisfaction), and (c) SQ 3 (Function) in comparison with the Banff Patella Instability Instrument 2.0 (BPII 2.0). SQ, Single Assessment Numeric Evaluation question.

Compared with the pre‐operative data, the post‐operative correlations between BPII 2.0 and SQ1, SQ2 and SQ3 changed from high to low for SQ1 (*r* = 0.23; 95% CI, −0.13 to 0.53; *p* = 0.2), from low to moderate for SQ2 (*r* = 0.61; 95% CI, 0.32–0.79; *p* = 0.0003), and from low to high for SQ3 (*r* = 0.84; 95% CI, 0.70–0.92; *p* < 0.0001). Post‐operative Bland–Altman analysis between BPII 2.0 and SQ1, SQ2 and SQ3 revealed biases of −2.2 (SD, 19.3), −0.4 (SD, 15.4) and −4.8 (SD, 10.8), respectively (Figure 1). No redislocation occurred in any of the patients during the follow‐up period, as reported by the patients. There were no operation‐specific complications, and no reoperations were performed except for the planned removal of hardware.

## DISCUSSION

The main results of this study indicate that all three SQs reflected significant post‐operative improvement, which is consistent with BPII 2.0 outcomes. However, the correlation strength significantly varied from pre‐ to post‐operative. While SQ1 (Instability) demonstrated concurrent validity with BPII 2.0 pre‐operatively, SQ3 (Function) exhibited the strongest correlation with BPII 2.0 post‐operatively. These findings suggest that symptoms of LPIs may become less relevant after successful patellar‐stabilising procedures, whereas perceived overall knee joint function appears to have greater importance for disease‐specific quality of life.

The rationale for incorporating SANE into the present study was to determine whether a simple, global single‐item instrument can validly reflect disease‐specific quality of life in patients with lateral patellar instability. SANE captures the patient's overall perception of joint function using a single numeric rating, thereby minimising administrative burden while providing a quantitative estimate of perceived joint status [5, 14]. Previous investigations across multiple anatomical regions have demonstrated moderate to strong correlations between SANE and established multi‐item PROMs [15, 22, 24]. In shoulder populations, SANE has been shown to be significantly associated with instruments such as the American Shoulder and Elbow Surgeons (ASES) score [24]. In knee surgery, particularly after anterior cruciate ligament reconstruction, strong correlations between SANE and validated knee‐specific instruments, such as the IKDC and Lysholm scores, have been reported [22]. Moreover, O'Connor and Ring demonstrated consistent correlations between SANE and established PROMs across different orthopaedic conditions, concluding that SANE reflects overall patient‐perceived function despite its single‐item structure [15]. Collectively, these findings suggest that SANE performs comparably to more comprehensive instruments in capturing global functional status.

The rationale for performing patellar‐stabilising surgery includes facilitating an anxiety‐free return to activities of daily living and sports [13] and reducing the risk of patellofemoral osteoarthritis in the long term [21]. Because the impact of recurrent LPIs extends measurable physical deficits, the evaluation of treatment outcomes increasingly incorporates patient‐reported assessments of health‐related quality of life. To date, the BPII 2.0 is the most thoroughly validated instrument specifically developed to assess disease‐specific quality of life in individuals with LPIs [1, 28]. Despite favourable clinical outcomes and the absence of recurrent patellar dislocations, the post‐operative BPII score of 2.0 remained approximately 22 points below the maximal score of 100. This residual deficit also indicates that restoring patellar stability alone does not fully normalise disease‐specific quality of life in all individuals. These observations confirm previous evidence suggesting that redislocation rates alone are insufficient indicators of treatment success [13]. Prior studies have demonstrated that patients may discontinue sporting activities [18], report persistent subjective apprehension or instability [7], and experience fear of reinjury [10]. These results may reflect the shift in the correlation of the SANE question from instability‐driven symptoms pre‐operatively to functionality‐driven complaints post‐operatively in this study.

To further improve the clinical interpretability of post‐operative BPII 2.0 values, a recent work has established a patient acceptable symptom state (PASS) threshold for BPII 2.0 at 62.4 points following patellar stabilisation surgery, allowing clinicians to classify results not only as statistically improved but also as acceptable from the patients' perspective [25]. These results confirm previous findings that the likelihood of achieving the MCID after surgical stabilisation of recurrent LPIs is primarily determined by pre‐operative BPII 2.0 values (calculated at 65.2 points) and subjective functional ratings [2]. Findings have indicated that baseline patient‐reported impairment strongly influences the probability of perceiving post‐operative improvements [2]. In the cohort in this study, 83% of patients achieved the PASS threshold reported by Waaler et al. [25].

Recent evidence suggests that residual limitations in disease‐specific quality of life may be substantially influenced by psychological rather than purely physical determinants [9]. Hiemstra et al. demonstrated that kinesiophobia and pain catastrophizing are significantly associated with worse disease‐specific quality of life in patients with recurrent patellofemoral instability, suggesting that fear‐related cognitions and maladaptive pain‐related beliefs may persist even when mechanical instability is addressed surgically [9]. Extending this concept to post‐operative outcomes after medial patellofemoral ligament reconstruction (MPFL‐R), Rahn et al. reported that patient‐specific psychological characteristics—including self‐efficacy, pain catastrophizing, exercise‐related fear, and personality traits such as neuroticism—correlate with functional outcomes and psychological readiness to return to sport. These findings may be potential explanatory factors supporting the notion that post‐operative BPII 2.0 outcomes may reflect a broader biopsychosocial construct, including psychological readiness and perceived capability, rather than knee stability alone [19].

Recent research established age‐ and sex‐specific reference values for the BPII 2.0 in the German general population, indicating that reduced post‐operative BPII 2.0 scores cannot be attributed to limitations in knee joint function alone. The normative BPII 2.0 score averaged 79.6 ± 20.5 points in individuals aged 14–30 years [28], a range that closely aligns with the post‐operative values observed in the present cohort. The reference study also demonstrated that increasing age, female sex, comorbidities and elevated body mass index significantly diminish BPII 2.0 scores [28], indicating that patient‐specific factors beyond knee joint function and patellar stability influence BPII 2.0 values.

In addition to interpreting outcomes via the MCID and PASS, improvement thresholds linked to surgeon‐defined ‘satisfactory outcomes’ provide another clinically meaningful perspective on post‐operative success. Patel et al. demonstrated that the magnitude of improvement (MOI) in patient‐reported outcomes after isolated MPFL‐R was associated with satisfactory surgeon‐defined results. Notably, BPII 2.0 showed good discriminative ability for differentiating satisfactory from unsatisfactory outcomes, supporting its relevance for capturing domains beyond instability alone, such as sport‐specific confidence and perceived readiness [16]. This further substantiates the present finding that post‐operative outcome assessment should place greater emphasis on functional recovery and activity‐related participation rather than redislocation alone.

This study contributes to the literature by performing a post‐operative evaluation of the previously proposed single‐assessment numeric evaluation developed for patients with patellofemoral instability [6]. The strengths of the investigation include the prospective study design, relatively large patient cohort with homogenous subgroups concerning demographics, and use of the validated BPII 2.0 score. Nevertheless, certain limitations should be acknowledged. The patients included in this study were randomised into three groups on the basis of their demographic characteristics. At the time of randomisation, the specific surgical procedures required to address patellofemoral instability had not yet been determined. As shown in Table 1, the distribution of surgical procedures differed among the SQ1, SQ2 and SQ3 groups. This non‐significant heterogeneity in surgical management may have influenced the study results and is therefore considered a limitation. However, the correction of modifiable risk factors led to similarly outcomes in patients who underwent isolated MPFL reconstruction considering the BPII 2.0 and subjective assessment of knee joint function [29]. Moreover, further work is needed to confirm the construct and content validity of the assessment through additional testing. Owing to the study design, no conclusions can be drawn regarding intra‐ or interobserver reliability. Additionally, test‐retest reliability and responsiveness were not assessed. While SQ3 appears suitable as a screening tool for assessing disease‐specific quality of life post‐operatively, it is insufficient for drawing conclusions regarding therapeutic decisions in patients with post‐operative complaints. Rather, the SQ3 may be useful in easily identifying patients who continue to experience reduced disease‐specific quality of life after surgery. Importantly, the underlying cause of post‐operative impairment in quality of life does not necessarily originate from the treated knee joint alone but may also be influenced by other patient‐specific factors. In line with emerging evidence on the psychological determinants of outcomes after MPFL‐R, the SQ3 may therefore serve as a pragmatic screening tool to identify patients who could benefit from additional multidimensional evaluation, including assessment of fear of reinjury, pain catastrophizing, self‐efficacy, and readiness to return to sport [9, 19]. Considering that the PASS and MOI thresholds for BPII 2.0 are now available, future studies should also evaluate whether SQ3 can accurately identify patients who fail to reach an acceptable symptomatic state or surgeon‐defined satisfactory outcomes, thereby facilitating targeted post‐operative support and shared decision‐making [16, 25].

## CONCLUSION

All three SANE questions reflected significant post‐operative improvement, which was consistent with BPII 2.0 outcomes. However, correlation and measurement biases varied from pre‐ to post‐operative. While SQ1 (Instability) demonstrated pre‐operative concurrent validity with BPII 2.0, as previously shown, SQ3 (Function) exhibited the strongest correlation with BPII 2.0 post‐operatively, warranting cautious interpretation given study limitations.

## AUTHOR CONTRIBUTIONS

**Felix Zimmermann**: Study design; data collection; data analysis; data interpretation; writing the paper; final approval. **Julian Flügel** and **Linda Biester**: Study design; data collection; data analysis; final approval. **Sebastian Gebhardt**, **Paul Alfred Grützner**, and **Emmanouil Liodakis**: Data analysis; data interpretation; final approval. **Peter Balcarek**: Study design; data analysis; data interpretation; writing the paper; final approval.

## CONFLICT OF INTEREST STATEMENT

The authors declare no conflicts of interest.

## ETHICS STATEMENT

The study was conducted in accordance with the Declaration of Helsinki and approved by the local ethics committee Baden‐Württemberg, Germany (F‐2019‐070). Informed consent was obtained from all the subjects involved in the study.

## Data Availability

The data sets used and analysed during the current study are available from the corresponding author on reasonable request.
